# Impact of Long-Term Shaolin Zen Meditation on Emotional Processing in Aging: A Visual ERP Study

**DOI:** 10.3390/bs13060513

**Published:** 2023-06-19

**Authors:** Huang Gu, Tiantian Liang, Zhaoyang Xin, Zilu Lu, Qiaoling Li, Hao Hong

**Affiliations:** 1 Institute of Behavior and Psychology, School of Psychology, Henan University, Kaifeng 475004, China; 10030106@vip.henu.edu.cn (H.G.); tian2023@henu.edu.cn (T.L.); psyxin2021@henu.edu.cn (Z.X.); mellowlu@163.com (Z.L.); 2 Zhongyuan Wushu Research Institute, Henan University, Kaifeng 475000, China

**Keywords:** Shaolin Zen meditation, emotion, cognition, aging, ERP

## Abstract

The aging process is always accompanied by a decline in cognitive and emotional functions. Although previous studies have identified the positive effects of different meditative practices on emotional and cognitive functions, few studies have investigated the most primitive Chinese meditation—Shaolin Zen meditation. In particular, data are extremely limited regarding the brain mechanism of the effects of Shaolin Zen meditation on cognitive and emotional functions during aging. The current study aimed to explore the effects of long-term Shaolin Zen meditation practice on event-related potentials (ERPs) during facial emotion recognition in aging. ERPs were recorded from 16 monks with long-term meditation experience and 20 controls without meditation experience. The significant age-related degenerative changes in the early ERP components did not present in the meditators but only in the controls without meditation experience. Additionally, we found no group differences in the late P3 component. These findings suggest that long-term Shaolin Zen meditation practice can counteract the age-related cognitive decline in the “down-top” automatic processing of emotional stimuli.

## 1. Introduction

Meditation is a form of self-regulatory exercise for the mind and body which highlights paying attention to the present moment and attaining a state of consciousness in an accepting manner [1]. The positive effects of meditation on human mental and physical health have been well documented, especially in terms of relieving stress, enhancing positive emotions such as love and well-being, and protecting against cognitive decline at an advanced age [2,3,4]. Meanwhile, growing evidence has revealed the clinical use and efficacy of the underlying neurobiological mechanisms. Specifically, meditation practice contributes to improving coping with chronic diseases of aging [5,6], neurodegenerative diseases such as Alzheimer’s disease [7], and mental disorders, i.e., major depression diseases, addiction disorders, and schizophrenia [8,9,10]. Recently, the emotional and cognitive effects of different meditative practices have received significant attention from researchers interested in preventing emotional and cognition disorders and promoting healthy aging in novel and less invasive ways.

Aging is a natural process that is associated with the decline in cognitive and emotional functions [11,12] and the emergence of mental and physical diseases [13,14,15]. The emotional problem in particular, as a critical aspect of aging, has been shown to occur both in normal aging and pathological conditions [16]. For instance, emotional dysregulation and maladaptive emotion regulation have led to the high prevalence of psychiatric disorders such as anxiety disorders and depression among middle-aged and older people [17]. In addition, many studies have shown that older adults have difficulties in recognizing facial anger, sadness, and fear [18] and are different from young adults in the brain activation induced by different emotional faces [19,20]. Overall, these emotional disorders may lead to many social dysfunctions, including impaired social competence and decreased social interest, thus affecting the quality of life of middle-aged and elderly people.

Recently, growing research on the emotional and cognitive effects of meditation has confirmed that long-term meditation seems to be efficient in delaying aging and improving emotion [4,21,22]. As several original studies showed, long-term meditation practice can reduce negative emotionality, relieve mental tension and stress, and offset age-related cognitive decline [4,23,24,25]. Further, neuroscience research has deepened the understanding of the underlying mechanisms of the emotional effects of meditation. These studies have focused on the late positive potential component (LPP), which is seen as a neurophysiological marker of sensitivity to the valence of emotional stimulus and reflects high levels of processing of emotional stimuli [26,27]. For example, an event-related potential (ERP) study conducted by Sobolewski et al. found that long-term meditation practitioners showed smaller LPPs elicited by negative stimuli compared with non-meditating controls. They argued that meditators are less affected by stimuli with an adverse emotional load at high levels of processing [28]. Consistent with this finding, multiple neuroimaging studies have revealed that long-term meditation training is associated with increased prefrontal activation and decreased amygdala activation in response to emotional stimuli [29,30,31,32].

Previously, many studies have explored various forms of commonly known meditation practice, such as mindfulness meditation, mindfulness-based stress reduction (MBSR), Yoga meditation, and Zen meditation [29,32,33,34]. However, few studies have investigated the most primitive Chinese meditation—Shaolin Zen meditation, which is a core element of Shaolin culture [35] and has very distinct characteristics. Instead of purely static meditation, it seeks to combine Buddhist health preservation with Shaolin’s internal skill, the essence of Zen with Shaolin’s mindfulness, and Zen meditation with Shaolin sitting meditation [36]. These characteristics of Shaolin Zen meditation described above have been suggested to promote healthy aging and emotional regulation, such as relieving stress, reducing symptoms of anxiety and depression, bringing the mind into a tranquil state, and preserving health [36,37,38]. Although Shaolin Zen meditation has been used for thousands of years since ancient times, few studies have explored its brain mechanism; in particular, the impact of Shaolin Zen meditation on emotional processing during the aging process has not, to our knowledge, been the subject of a visual ERP study.

Therefore, this study aimed to investigate the effect of long-term Shaolin Zen meditation practice on emotional processing in aging. To achieve this, monks with long-term meditation experience in Shaolin Temple were selected as the study objects, and the ERP technique was employed because its high time resolution facilitates the identification of the temporal characteristics of long-term Shaolin Zen meditators in the cognitive process of facial emotion recognition [28,33]. Based on the findings of previous ERP studies on facial emotion processing, we focused on three ERP components sensitive to facial emotion, including N1, N170, and P3 [39,40].

## 2. Materials and Methods

### 2.1. Participants

The experiment included 2 groups of healthy right-handed males: 16 experienced long-term meditation monks (meditators group) from Shaolin Temple in China and 20 age-matched healthy controls with no meditation experience (control group). The total number of participants was determined based on previous ERP studies of long-term meditators [28]. The meditators group had an average of 13.630 years of Shaolin Zen meditation experience and maintained a regular daily meditation schedule of 4:15–6:00 am, 9:10–11:00 am, 12:30–13:00, 15:40–17:40, and 19:30–20:30 pm. Characteristics of the meditators group and control group are summarized in Table 1. Both groups were male. All participants were native Chinese speakers and reported no history of somatic disorders or neurological diseases. All participants provided written informed consent and were paid for participation. The study protocol was approved by the Institutional Review Board of Henan University.

### 2.2. Task Design and Procedures

**Figure 1:**
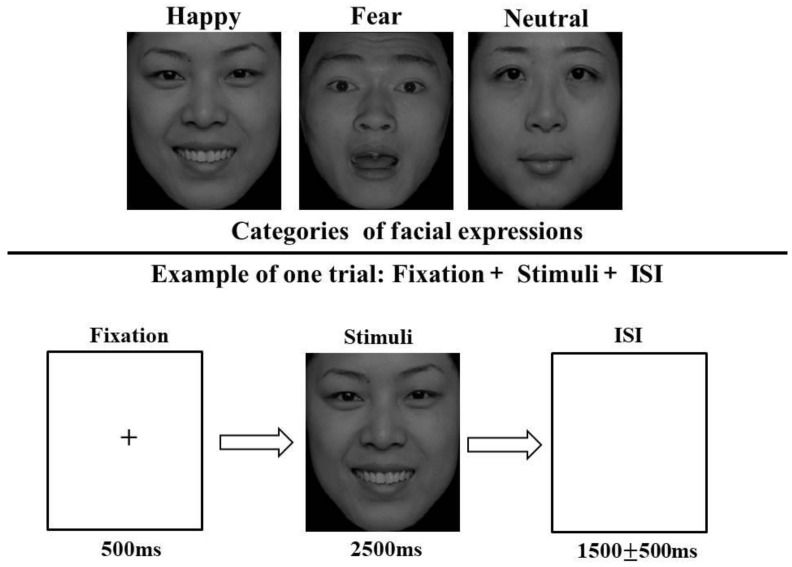
A trial of a facial emotion recognition task.

The stimulus images used in the study were selected from the Native Chinese Facial Affective Picture System (CFAPS), including 120 pictures (half male and half female) with different emotional valences (Happy, Fear, Neutral) [39]. There was no significant difference in arousal among the 3 emotional faces (*p* > 0.05). The participants were seated in a quiet room for the experiment, approximately 80 cm away from a computer screen with horizontal and vertical visual angles below 6. The entire experiment was divided into 2 blocks of 120 trials. To familiarize the participants with the task, the experiment began with 18 practice trials. In our main experiment, as shown in Figure 1, each trial began with the presentation of a fixation for 500 ms. Subsequently, 1 facial stimulus was presented for 2500 ms. Participants were instructed to make a correct judgment quickly for the facial expression by pressing response buttons (“4”, “5”, and “6”) corresponding to “Happy”, “Fear”, and “Neutral”, respectively. The facial stimulus disappeared after the participants responded to it. The inter-stimulus interval (ISI) lasted for 1500 ± 500 ms, during which the screen was kept white and blank. One-minute rest was allowed between blocks so as to avoid fatigue.

### 2.3. Electrophysiological Recording and Preprocessing

EEG was performed from 32 scalp sites using tin electrodes mounted in an elastic cap (Brain Products; Munich, Germany) according to the extended 10–20 system, each referenced online to FCz [41]. To monitor ocular movements and eye blinks, electrooculogram (EOG) recordings were taken from electrodes located below the right eye. Electrode impedance was maintained below 10 kΩ. The EEG and EOG signals were amplified using a 0.05–100 Hz online band-pass filter and continuously sampled at 1000 Hz/channel for offline analysis.

The EEG data were preprocessed using Analyzer 2.1. First, the data were resampled to 250 Hz and then low-pass-filtered at 30 Hz and band-stop-filtered to eliminate 50 Hz line noise. The continuous data were re-referenced to the algebraic average of the electrodes at the left and right mastoids to better detect signals from areas along the midline. An independent component analysis (ICA) algorithm was then performed to correct artifacts relevant to eye blinks and movements [42,43]. The ocular-artifact-corrected EEG signal was then segmented starting at 200 ms before stimulus presentation through 800 ms with the first 200 ms pre-stimulus as baseline (−200 ms–0 ms). The baseline was corrected using whole epochs to improve the reliability of the independent components. Trials in which the EEG voltage exceeded a threshold of ±80 μV were excluded from further analysis.

### 2.4. ERP Analysis

This study analyzed ERPs elicited by stimulus presentation. The electrodes and time window for each ERP component were determined based on previous research and the visual detection of grand-averaged waveforms and the scalp distribution. Namely, we analyzed the peak amplitudes of the N1 (100–200 ms) and N170 (140–240 ms) components at P3/P4/Pz [44] and P7/P8 [39,45], respectively. The mean amplitude of the P3 component (340–460 ms) at C3/C4/Cz [40] was analyzed.

### 2.5. Statistical Analysis

This study focused on the interaction between Age and Meditation. Therefore, we used Age as the independent variable and categorized subjects below or equal to 40 years old into the younger group and those above 40 years old into the older group. The age ranges obtained by using age 40 as the separator matched those in previous studies [19,46]. An independent sample *t*-test was used to analyze the demographic characteristics of the groups. A repeated-measure analysis of variance (ANOVA) with Meditation (meditators/control group) and Age (younger/older group) as between-subjects factors and facial expression (Happy/Fear/Neutral) as a within-subjects factor to analyze the accuracy rate, reaction time, and ERP (peak amplitudes of N1 and N170 components and mean amplitude of P3 component) data, respectively, under SPSS 21.0. Further, a two-tailed Pearson’s r correlation was used to determine the correlation between the peak amplitude of the N1 component and age and examine the relationship between the negative activation of the N1 component and age. Descriptive data are presented as arithmetic mean (M) ± standard error (SD). The degrees of freedom of the *F*-ratio were corrected according to the Greenhouse–Geisser method. Bonferroni correction was applied to all post hoc tests.

## 3. Results

### 3.1. Behavioral Results

Table 2 shows the response time (RT) and accuracy (ACC) means and standard errors for three emotional expressions in the meditators group and control group. Accuracy was significantly affected by Emotion (*F*_(2,58)_ = 7.556, *p* = 0.002, *η*^2^ = 0.207). The post hoc comparison showed that the ACC for happy expressions (0.969 ± 0.007) was higher than for fearful (0.914 ± 0.015) or neutral expressions (0.930 ± 0.011).

The response time was significantly affected by Emotion (*F*_(2,58)_ = 8.276, *p* = 0.001, *η*^2^ = 0.222). The post hoc comparison showed that the RT for happy expressions (860.325 ± 26.535) was faster than for fearful (960.411 ± 27.188) or neutral expressions (947.925 ± 24.401). The response time was also significantly affected by Age (*F*_(1,29)_ = 4.895, *p* = 0.035, *η*^2^ = 0.144). The post hoc comparison showed that the RT for the younger group (876.481 ± 30.655) was faster than for the older group (969.293 ± 28.638).

### 3.2. ERP Results

- N1

**Figure 2:**
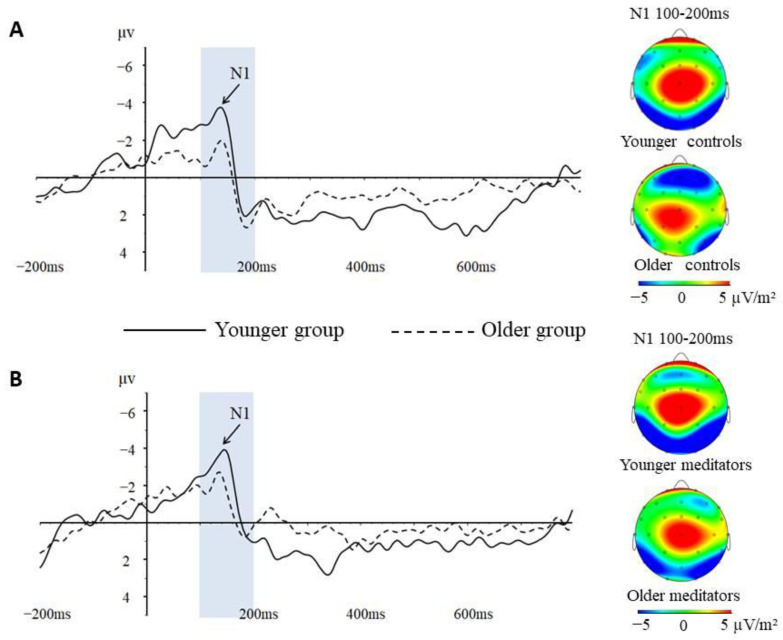
(**A**) N1 component at Pz electrode and topographic maps of the N1 scalp voltage of controls; (**B**) N1 component at Pz electrode and topographic maps of the N1 scalp voltage of meditators.

The repeated-measures ANOVA revealed a marginally significant main effect of Emotion (*F*_(2,29)_ = 2.991, *p* = 0.058, *η*^2^ = 0.091). Follow-up comparisons displayed a higher N1 amplitude for fearful expressions (−3.760 ± 0.337) when compared with neutral expressions (−3.280 ± 0.311), whereas there were no significant differences among the remaining facial expressions (*p* > 0.05). The effect of Meditation was also marginally significant (*F*_(1,30)_ = 3.566, *p* = 0.069, *η*^2^ = 0.106). The meditators (−4.078 ± 0.450) showed a larger N1 amplitude compared with the controls (−2.967 ± 0.380). The Meditation × Age interaction was significant (*F*_(1,30)_ = 5.886, *p* = 0.021, *η*^2^ = 0.164) (Table 3). The follow-up repeated-measures ANOVA (Age) was calculated for each group separately. In the control group, there was a significant main effect of Age (*F*_(1,18)_ = 11.629, *p* = 0.003, *η*^2^ = 0.392); the younger controls (−4.105 ± 0.517) showed a larger N1 amplitude compared with the older controls (−1.828 ± 0.422) (Figure 2A). In contrast, the Age effect was insignificant in the meditators group (Figure 2B).

**Figure 3:**
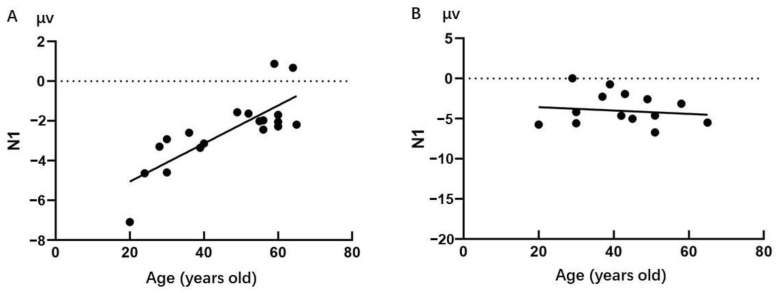
(**A**) The peak amplitude of N1 component and age in control group; (**B**) the peak amplitude of N1 component and age in meditators group; ●: a participant.

Additionally, the correlational analysis showed that in the control group the peak amplitude of the N1 component was significantly correlated with age (*r* = 0.703, *p* = 0.001) (Figure 3A). With an increase in age, the amplitude of the N1 component decreased significantly. While in the meditators group, there was no significant correlation between the peak amplitude of the N1 component and age (*r* = −0.125, *p* = 0.669) (Figure 3B). With an increase in age, the amplitude of the N1 component did not change significantly.

- N170

**Figure 4:**
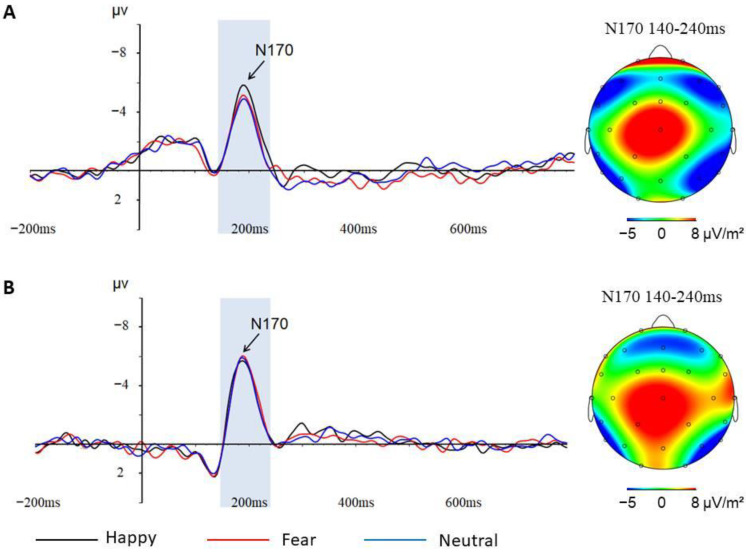
(**A**) N170 component at P8 electrode and topographic maps of the N170 scalp voltage of younger controls; (**B**) N170 component at P8 electrode and topographic maps of the N170 scalp voltage of older controls.

**Figure 5:**
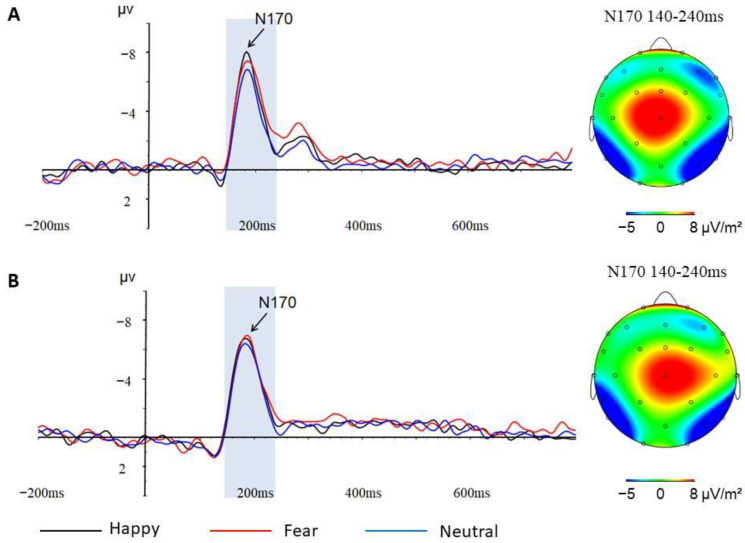
(**A**) N170 component at P8 electrode and topographic maps of the N170 scalp voltage of younger meditators; (**B**) N170 component at P8 electrode and topographic maps of the N170 scalp voltage of older meditators.

The repeated-measures ANOVA on the N170 amplitude revealed a significant effect of Meditation (*F*_(1,30)_ = 4.209, *p* = 0.049, *η*^2^ = 0.120). The meditators (−7.446 ± 0.719) showed a larger N170 amplitude compared with the controls (−5.432 ± 0.668). The Emotion × Meditation interaction was also significant (*F*_(2,33)_ = 4.430, *p* = 0.019, *η*^2^ = 0.125). The simple effects analyses revealed that the main effect of Meditation was significant for happy and fearful expressions (*F*_(1,33)_ = 4.543, *p* = 0.041, *η*^2^ = 0.121) (*F*_(1,33)_ = 5.887, *p* = 0.021, *η*^2^ = 0.151): the meditators (−7.512 ± 0.718) (−7.732 ± 0.721) showed a larger N170 amplitude compared with the controls (−5.436 ± 0.659) (−5.359 ± 0.661). In contrast, the Meditation effect was insignificant for neutral expressions (*F*_(1,33)_ = 3.043, *p* = 0.090, *η*^2^ = 0.084). The Emotion × Meditation × Age interaction was also significant (*F*_(2,33)_ = 4.128, *p* = 0.024, *η*^2^ = 0.118) (Table 3). The further simple effects analyses demonstrated that there was a significant interaction between Emotion and Age in the control group (*F*_(2,33)_ = 3.793, *p* = 0.041, *η*^2^ = 0.182): the significant Emotion effect did not occur in the older controls but only in the younger controls (*F*_(2,16)_ = 4.749, *p* = 0.024, *η*^2^ = 0.373) (Figure 4). In contrast, the interaction of Emotion and Age was insignificant in the meditators group (*F*_(2,33)_ = 1.285, *p* = 0.290, *η*^2^ = 0.084), but the meditators showed a significant Emotion effect (*F*_(2,28)_ = 5.218, *p* = 0.017, *η*^2^ = 0.272) (Figure 5).

- P3

The repeated-measures ANOVA on the P3 amplitude revealed a significant effect of Emotion (*F*_(2,28)_ = 3.828, *p* = 0.030, *η*^2^ = 0.117), while the effects of Meditation and Meditation × Emotion and Meditation × Age were insignificant (Table 3). Fearful faces (1.085 ± 0.177) elicited a P3 that was greater than responses to happy (0.832 ± 0.173) and neutral ones (0.808 ± 0.174), and there was no significant difference between happy and neutral faces (*p* > 0.05).

## 4. Discussion

The present study aimed to explore how facial emotion recognition processing changes with age and whether the age-related changes would be influenced by long-term Shaolin Zen meditation. The result of this study showed that N1 and N170 components demonstrated important changes with increasing age, which revealed age-related cognitive decline during emotional face processing.

### 4.1. The Aging Process of Facial Emotion Recognition Processing

Our results showed that the activation of the N1 and N170 components was very sensitive to increased age. With an increase in age, the activation of the N1 component decreased significantly and the Emotion effect on the N170 component also disappeared. However, significant age-related changes were only present in the control group. According to the hypothesis of three stages of facial expression processing proposed by Luo et al., the N1 and N170 components, as the neural index of the early and middle stages of facial expression processing, reflect the early perceptual detection of information and the configuration detection of faces, respectively [39]. Thus, the degenerative changes we found on the N1 and N170 components may represent a decline in early attention and structural encoding abilities of emotional faces in older adults. This finding is consistent with previous studies that demonstrated that aging has negative effects on cognitive and emotional functions [18]. For example, Virtanen et al. [18] found that the ability to recognize facial emotions was affected at an early stage of cognitive impairment in older adults. In short, the current study found significant age-related degenerative changes occurring on the N1 and N170 components only in controls without Shaolin Zen meditation experience, which suggested that the cognitive decline in the elderly is closely linked to the abilities of early detection and structural encoding of emotional faces and that long-term Shaolin Zen meditation practice may have benefits on this age-related cognitive decline.

The current study did not observe a significant effect of age on the P3 component within the two groups. Similar results were reported in studies on the development of emotional face processing during childhood. Studies found that age and gender mainly influenced the early ERP components while showing few effects on the late components [47,48,49]. Thus, we also believe that aging had few significant effects on the late P3 component. In addition, the P3 component has generally been associated with task difficulty [39,50,51]. Therefore, it is also likely that the absence of an age effect on the P3 component in the present study was caused by the so-called “ceiling effect” stemming from the overly simple experimental task. Our behavioral results showed no significant age group differences in both accuracy and response time for facial emotion recognition, which indirectly supports our explanation.

### 4.2. The Positive Effect of Long-Term Shaolin Zen Meditation on the Aging Process

As we mentioned above, long-term Shaolin Zen meditation practice could indeed influence the aging process of emotional face processing. Our results indicated a significant interaction between Meditation and Age on the N1 component. The control group showed that older controls had significantly lower activation than younger controls, while in the meditators group, older meditators did not show significant differences from younger meditators. For the N170 component, the older controls showed an absence of the Emotion effect, which was present in the younger controls and the meditators group. Apparently, the control group showed significant age-related neurodegenerative changes in the early stage of facial emotion recognition processing, which is consistent with the general aging populations. In contrast, the meditators group did not show such significant degenerative changes with aging. In addition, we further confirmed this finding by using the correlation algorithm. The result showed that a significant negative correlation between age and activation of the N1 component did not occur in the meditators group but only in the control group. It was evident that the relative sparing of cognitive functions with age in the meditators group resulted in a lack of age effects.

Previous studies have concluded that the cognitive decline experienced by older adults during the early stage of emotional processing primarily reflected the aging of the automatic processing of emotional stimuli [18]. Thus, the relative sparing of cognitive functions with age in meditators may indicate that long-term Shaolin Zen meditation could intervene to some extent in the aging process that occurred during the automatic processing stage of emotional stimuli. According to previous studies, long-term meditation practice does have beneficial effects on the aging process [52,53]. For instance, studies on the impact of meditation on cognition in aging have found a positive effect of meditation training on cognitive functions, especially on attention and memory, known to be particularly sensitive to aging [54,55]. In conclusion, in terms of the technical aspect, the present study has once again demonstrated that long-term meditation played an important role in slowing down the aging process.

The present study did not observe the significant effect of meditation on the P3 component in the two groups. Like the previous study examining the emotional processing in long-term Sahaja Yoga meditators, the effect of meditation did not reach significance on the late ERPs but on the components within earlier time windows (140–400 ms), implying a higher sensitivity to detecting changes in the automatic “bottom-up” processes in the long-term Sahaja Yoga meditators [33]. Processing within the earlier latency range mainly reflects automatic (“bottom-up”) stimulus discrimination mediating by the attentional state [56,57], whereas the latter segments of the affective ERP (i.e., P3 and LPP) are sensitive to “top-down” control modulation by concurrent cognitive tasks or emotional regulation [58,59]. Thus, we assumed that it is the better attentional state of the meditators group that enhanced their early detection of emotional faces, thereby counteracting the age-related cognitive decline in the automatic process of emotional stimuli [54]. It is well established that a stable and focused state of attention is a prerequisite for entering into meditation, and all meditation forms involve the management of attention [60], especially Shaolin Zen meditation, which requires the meditators to abandon all distracting thoughts to focus on the present moment [38]. Several electrophysiological studies have also found that long-term meditation practice causes meditators to develop a dynamic bottom-up attention pattern, which benefits the ability to effortlessly sustain attention in the present moment while disengaging from distractions [61,62,63,64]. Altogether, these results strongly suggested that long-term Shaolin Zen meditation experience, as a neuroprotective factor, can counteract age-related deterioration in the automatic processing of emotional stimuli.

## 5. Conclusions and Limitations

In conclusion, this study paid more attention to the earlier ERP components that showed sensitivity to emotions and discovered age-related neurodegenerative changes that occurred during the earlier stages of facial emotion recognition in controls. These results might suggest cognitive decline in the automatic process during facial emotion recognition in older adults. Fortunately, we found that long-term Shaolin Zen meditation practice could contribute to improving the age-related cognitive decline in the automatic process of emotional stimuli.

However, one limitation of this study is that the long-term Shaolin Zen meditators selected for this study had different years of meditation experience. In future research, we should conduct a cross-sectional study to explore the EEG characteristics of emotional processing in long-term Shaolin Zen meditators with different years of meditation experience. In addition, the study was conducted exclusively on male meditators, and the sample size was small. Hence, the study findings must be interpreted with caution. Furthermore, the research is a cross-sectional study. A further longitudinal study could be conducted to developmentally explore the effects of long-term Shaolin Zen meditation on emotional and cognitive functions in the elderly.

## Figures and Tables

**Table 1:** Characteristics of meditators and controls (M ± SD).

	Meditators Group (*n* = 16)	Control Group (*n* = 20)	*T* (*p*)
Age (years)	41.630 ± 11.558	47.080 ± 14.709	−1.212 (0.234)
Meditation Experience (year)	13.630 ± 6.937		

**Table 2:** Mean accuracy and reaction time of meditators and controls (M ± SD).

	Meditators Group (*n* = 14)	Control Group (*n* = 19)	*F*_Meditation(*p*)_	*F*_Emotion(*p*)_	*F*_Meditation × Emotion(*p*)_	*F*_Meditation × Age(*p*)_	*F*_Emotion × Age(*p*)_	*F*_Meditation × Age × Emotion(*p*)_
Accuracy								
Total	0.929 ± 0.012	0.947 ± 0.010	1.335 (0.257)	7.556 (0.002 **)	0.948 (0.381)	0.401 (0.531)	1.868 (0.171)	3.264 (0.055)
Happy	0.957 ± 0.011	0.981 ± 0.010						
Fear	0.896 ± 0.022	0.931 ± 0.019						
Neutral	0.932 ± 0.017	0.928 ± 0.015						
Reaction time								
Total	923.336 ± 31.661	922.438 ± 27.523	0.000 (0.983)	8.276 (0.001 **)	0.554 (0.563)	0.441 (0.512)	1.510 (0.231)	0.832 (0.432)
Happy	875.825 ± 40.052	844.826 ± 34.817						
Fear	947.913 ± 41.038	972.909 ± 35.674						
Neutral	946.271 ± 36.832	949.579 ± 32.018	▭	▭	▭	▭	▭	▭

** *p* < 0.01.

**Table 3:** ERP components for meditators and controls (M ± SD) (μv).

	Meditators Group (*n* = 14)	Control Group (*n* = 20)	*F*_Meditation(*p*)_	*F*_Emotion(*p*)_	*F*_Meditation × Emotion(*p*)_	*F*_Meditation × Age(*p*)_	*F*_Emotion × Age(*p*)_	*F*_Meditation × Age × Emotion(*p*)_
N1 (peak amplitude)								
Total	−4.078 ± 0.450	−2.967 ± 0.380	3.566 (0.069)	2.991 (0.058)	0.044 (0.957)	5.886 (0.021 *)	1.003 (0.373)	2.457 (0.094)
Happy	−4.049 ± 0.454	−3.005 ± 0.384						
Fear	−4.332 ± 0.515	−3.187 ± 0.435						
Neutral	−3.853 ± 0.474	−2.707 ± 0.401						
N170 (peak amplitude)								
Total	−7.446 ± 0.719	−5.432 ± 0.668	4.209 (0.049 *)	1.940 (0.157)	4.430 (0.019 *)	0.062 (0.806)	0.678 (0.498)	4.128 (0.024 *)
Happy	−7.512 ± 0.736	−5.483 ± 0.684						
Fear	−7.732 ± 0.737	−5.330 ± 0.685						
Neutral	−7.095 ± 0.709	−5.484 ± 0.659						
P3 (mean amplitude)								
Total	1.124 ± 0.237	0.692 ± 0.222	1.766 (0.194)	3.828 (0.030 *)	0.356 (0.690)	0.780 (0.384)	1.563 (0.220)	1.535 (0.225)
Happy	1.003 ± 0.253	0.660 ± 0.236						
Fear	1.296 ± 0.258	0.873 ± 0.241						
Neutral	1.073 ± 0.254	0.544 ± 0.238						

* *p* < 0.05.

## Data Availability

The datasets presented in this article are not readily available due to privacy or ethical restrictions.
